# Supplementary material for: Determining off-target effects of splice-switching antisense oligonucleotides using short read RNAseq in neuronally differentiated human induced pluripotent stem cells
Source: Hum Mol Genet. 2025 Oct 9;34(22):1912–25. doi: 10.1093/hmg/ddaf153 (PMC12581821; doi:10.1093/hmg/ddaf153)
Supplement: Supplemental_figures_and_tables_hmg_ddaf153 [file supplemental_figures_and_tables_hmg_ddaf153.docx]

**Supplementary figures**

**
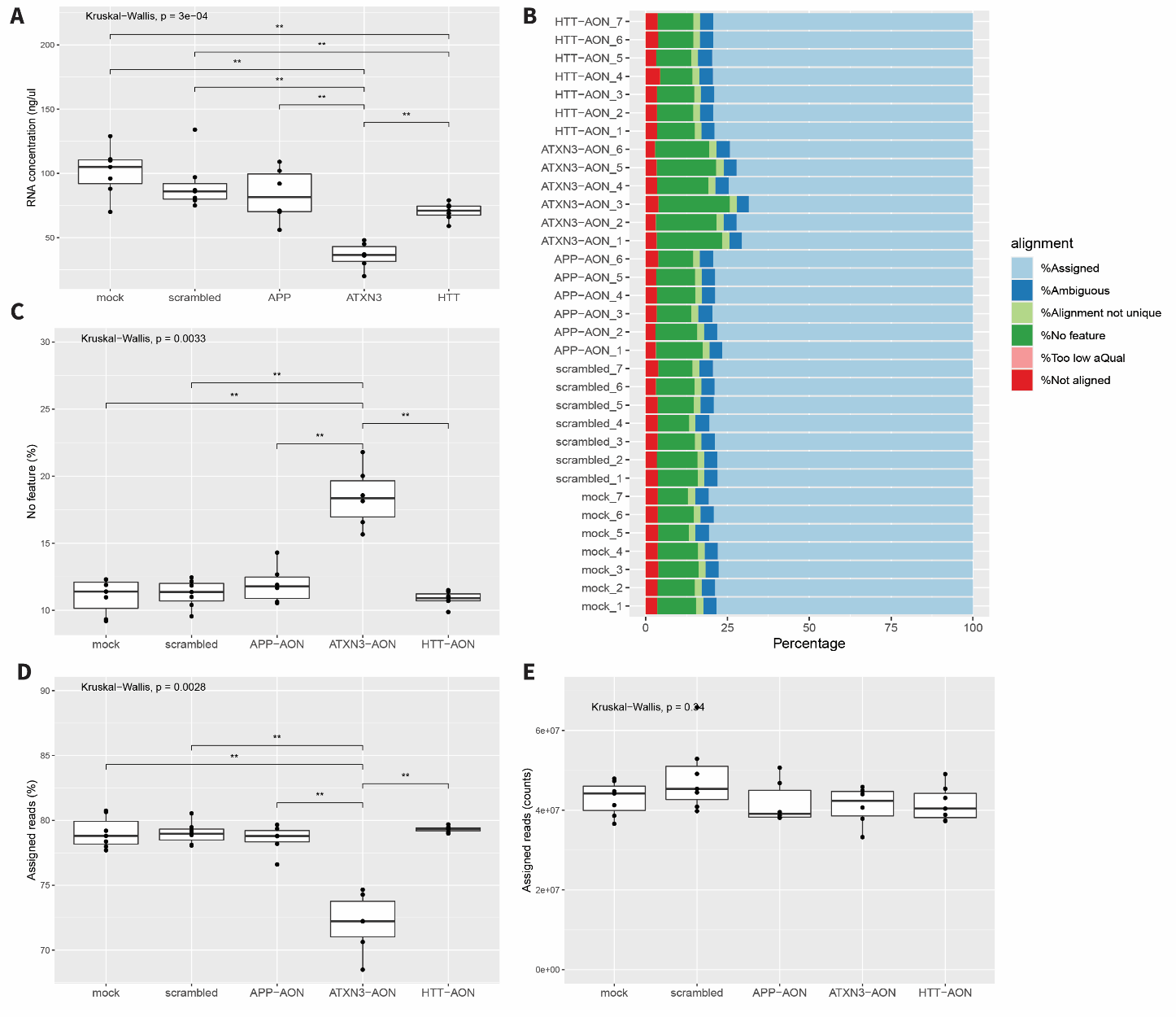
**

**Figure S1 Results of RNA isolation and RNA sequencing.** A) The RNA concentration upon isolation showed that especially transfection with the ATXN3-AON resulted in a lower RNA yield. B-D) Percentage-wise, more reads could not be assigned to a feature (C) and less reads were assigned to a gene (D) for ATXN3-AON-treated cells compared to the other conditions. E) Assigned reads per condition did not differ significantly between groups and showed an overall average of 43 million assigned reads. ** P < 0.01


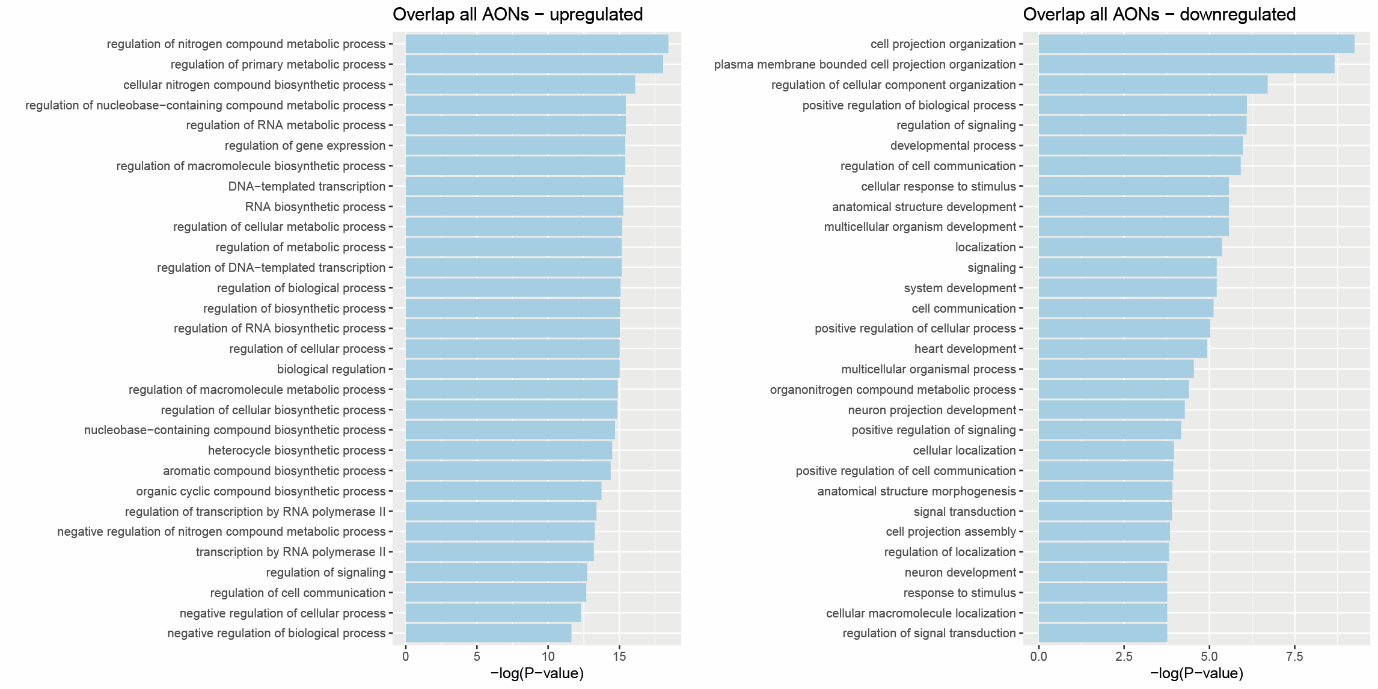


**Figure S2 Top 30 enriched pathways for the up- and downregulated genes shared by all three targeting AONs.**


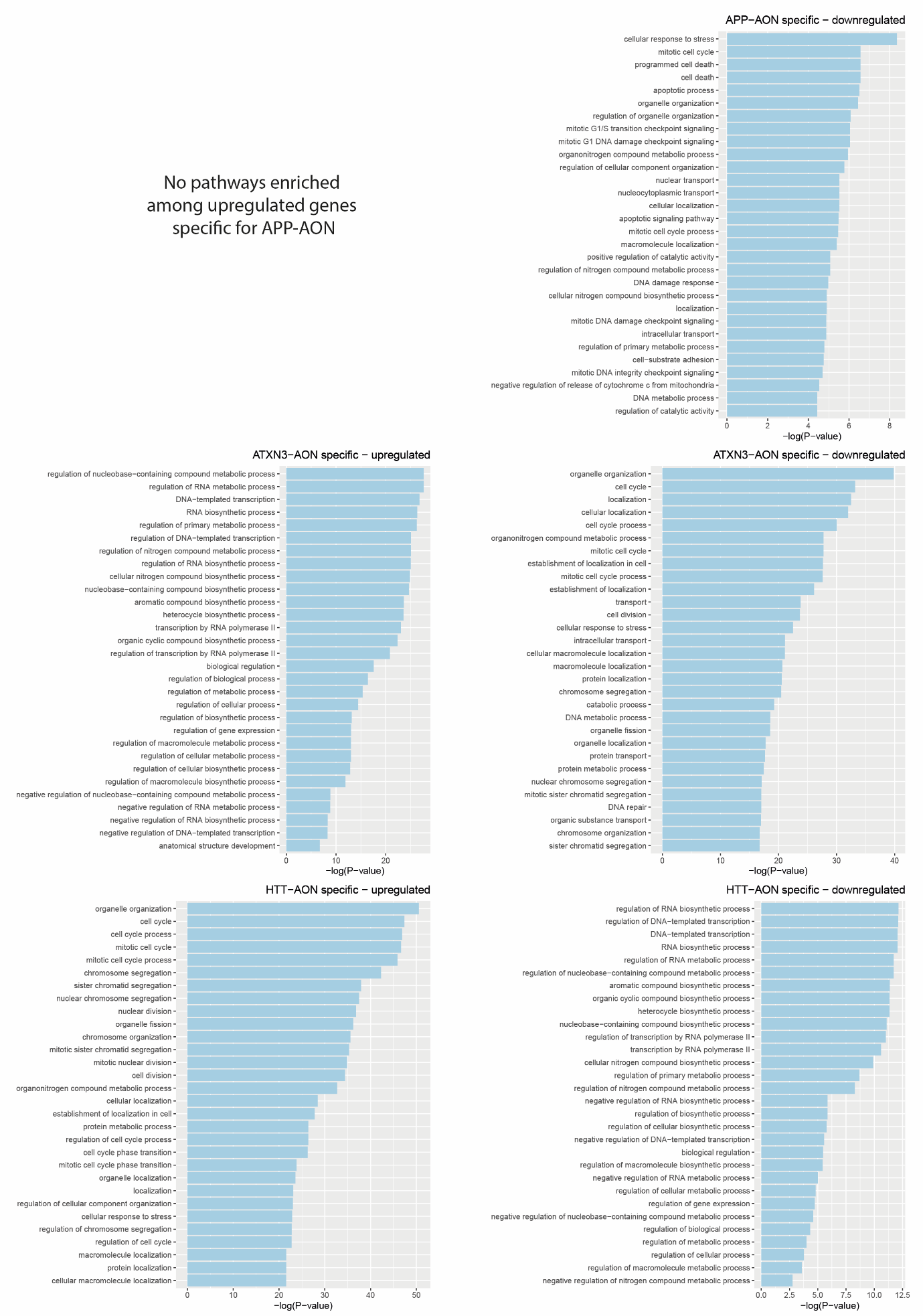


**Figure S3 Top 30 enriched pathways for the up- and downregulated genes specific for each targeting AON.**


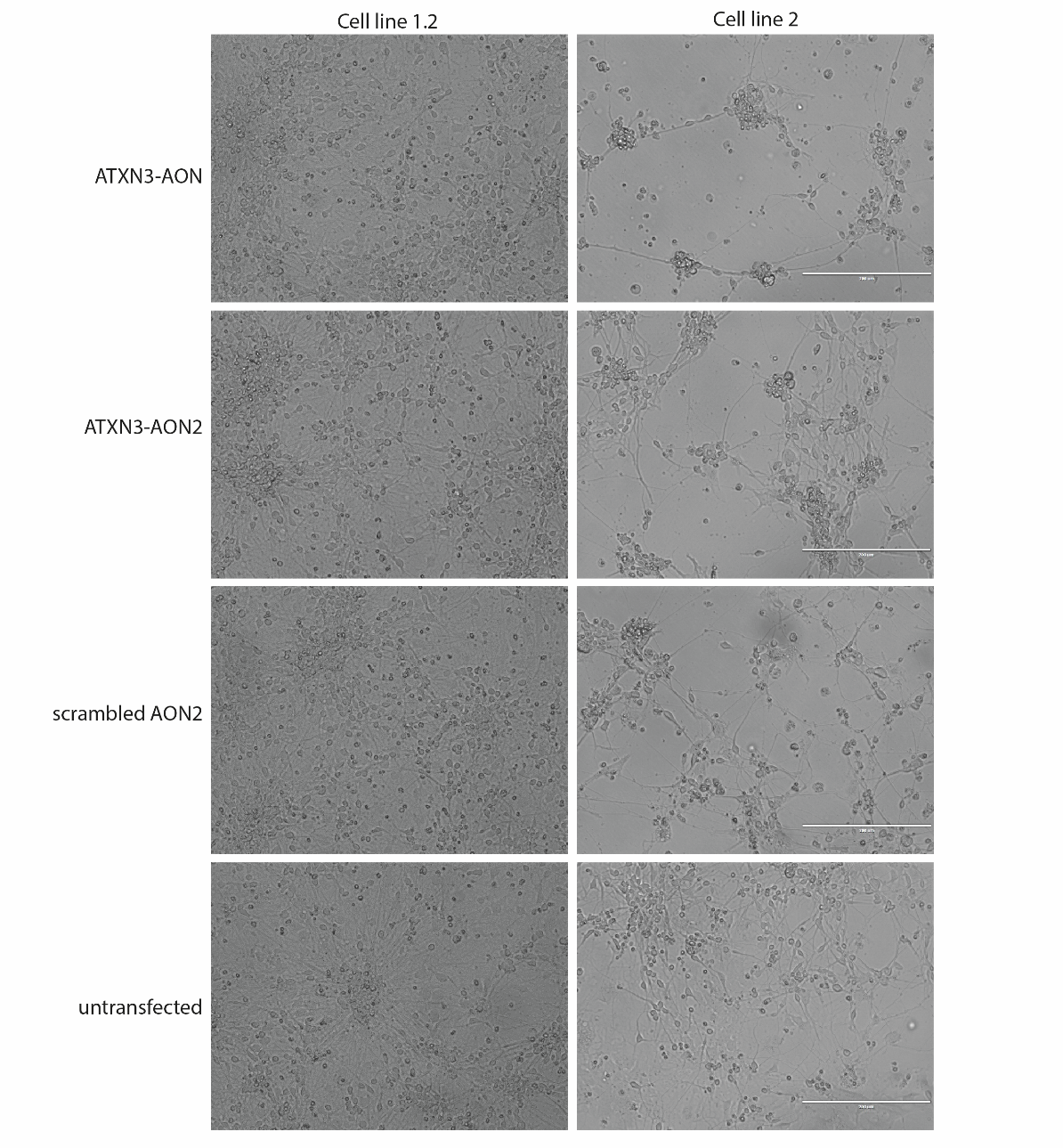


**Figure S4 Pictures of iPSC-derived neurons 3 days after transfection.** Especially transfection of ATXN3-AON led to cell death in cell line 2. Scale bar 200 µm; 20x magnification.


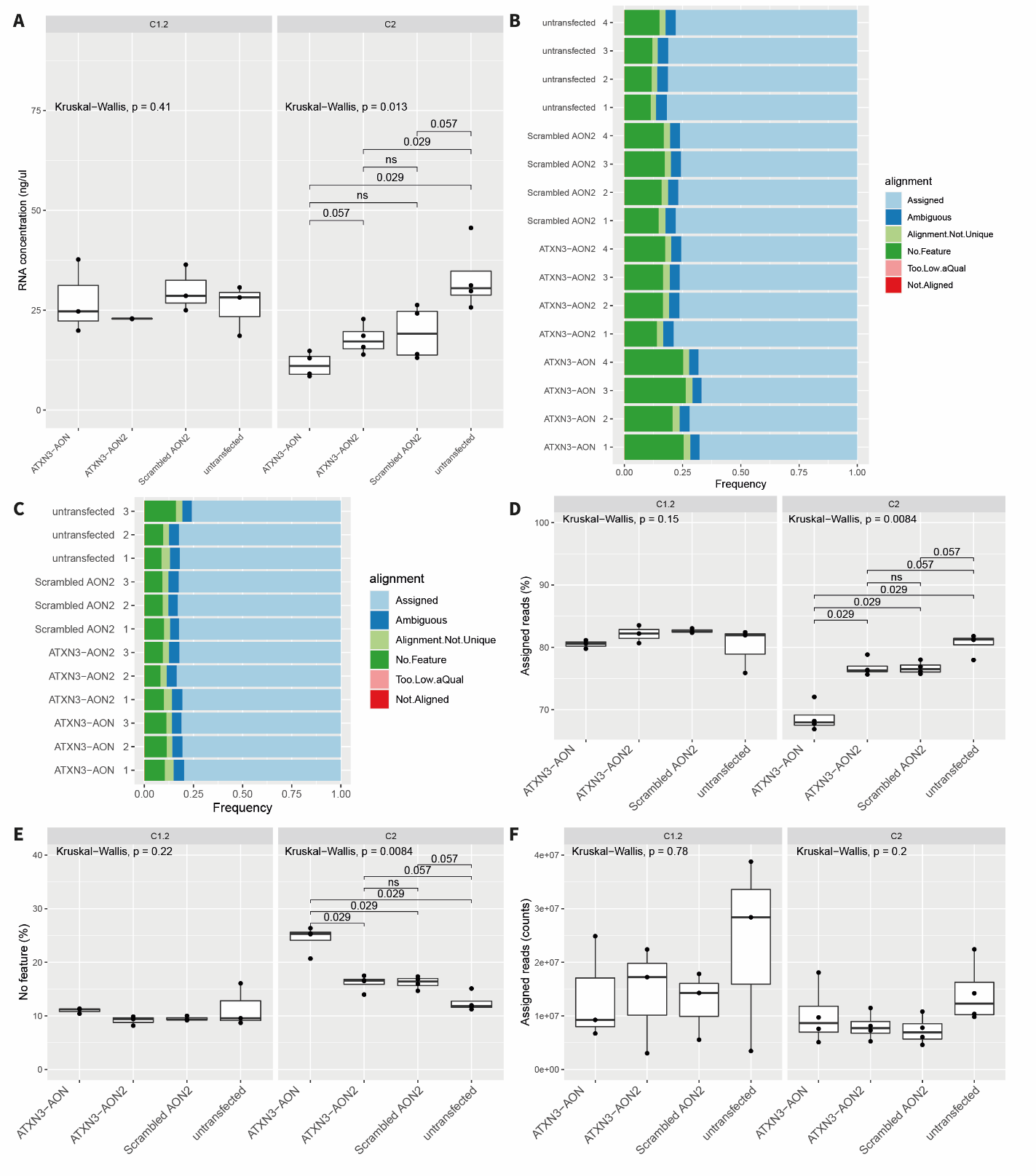


**Figure S5 Results of RNA isolation and RNA sequencing in cell lines C1.2 and C2.** A) Although the RNA concentration did not differ among the conditions in C1.2, especially transfection with the ATXN3-AON resulted in a lower RNA yield in C2. B) The alignment of reads showed less reads assigned to a gene and more reads showing no feature for ATXN3-AON-treated cells in C2 percentage-wise. C) This pattern was less obvious in C1.2. D-E) Consistently, statistical analysis of the percentage of reads assigned (D) and reads showing no feature (E) showed no difference among groups in C1.2, but a significantly lower percentage of reads assigned and percentage of reads showing no feature in C2. F) The number of assigned reads did not differ significantly among the groups in both cell lines.


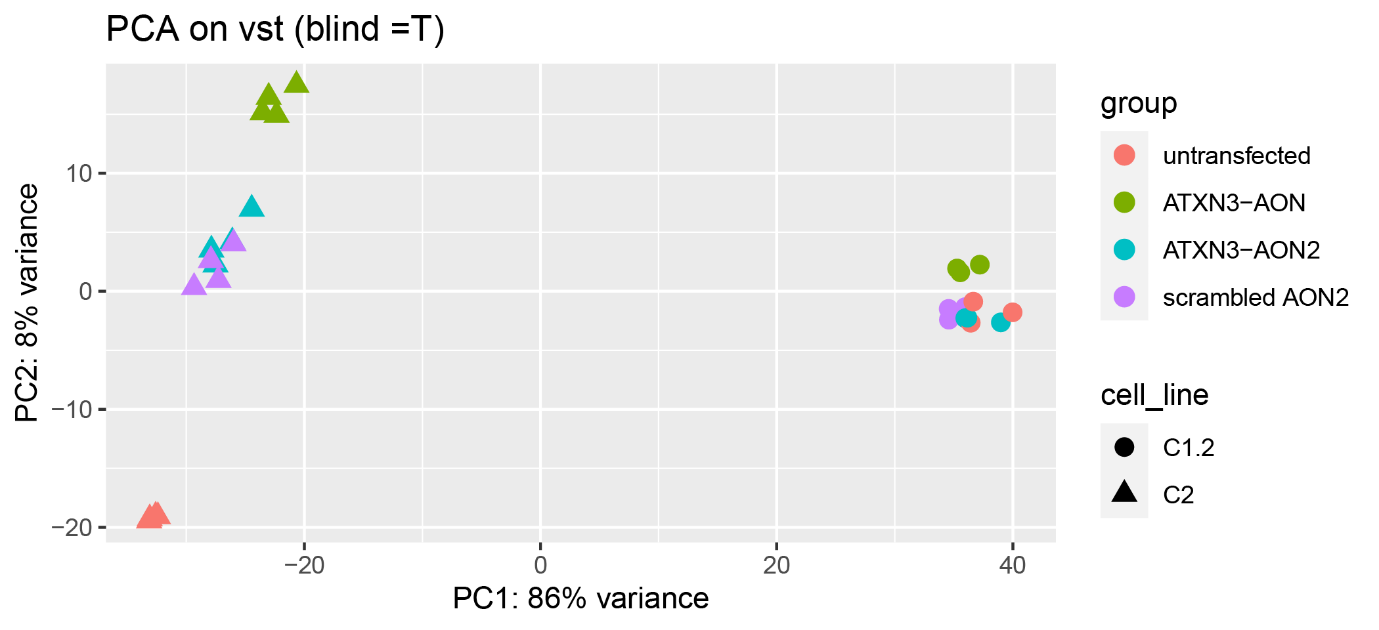


**Figure S6 Principle component analysis of the samples included in the validation experiment in cell lines C1.2 and C2.**


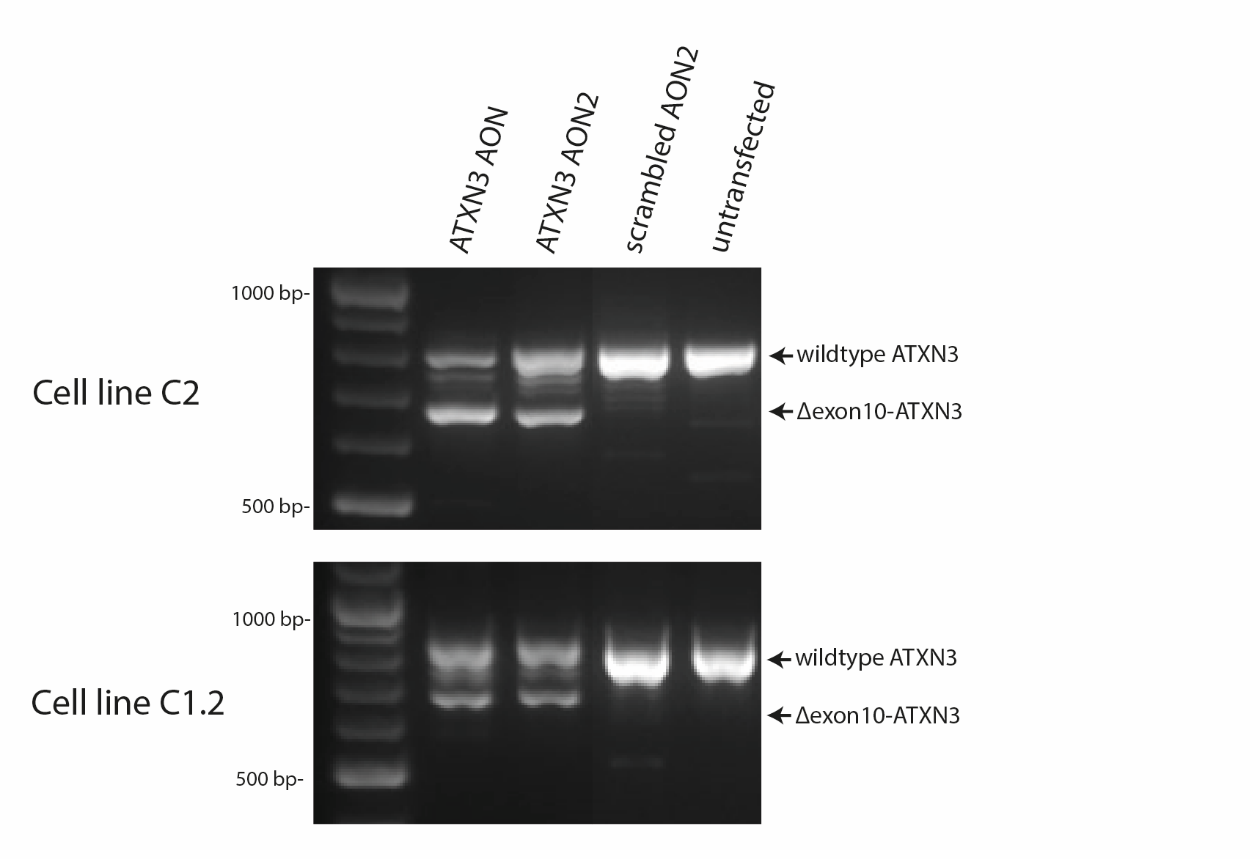


**Figure S7 The intended event in ATXN3 was confirmed by RT-PCR for both ATXN3-targeting AONs in both cell lines.**


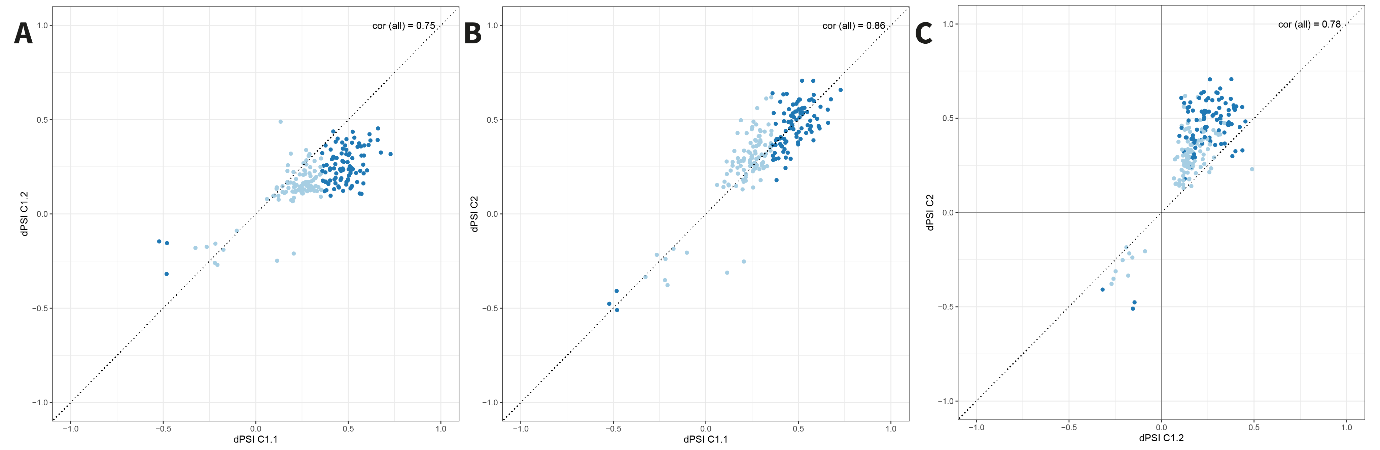


**Figure S8 Correlation of the shared differential splicing events induced by ATXN3-AON transfection when compared to scrambled AON (C1.1) or scrambled AON2 (C1.2 and C2).** dPSI: delta percent spliced in. AON-specific events larger than the intended event in ATXN3 identified in C1.1 are highlighted in darkblue. Correlations (cor) were determined using all shared events.

**Supplementary tables**

**Table S1 The significant events identified with rMATS and Whippet in the target genes represented the designed exon skip events.** SE: exon skipping; A5SS: alternative donor splice site; FDR: false discovery rate; PSI: percent spliced in; dPSI: delta PSI; Scr: scrambled AON; Prob: probability.

| **Scrambled AON vs. APP-AON** | | | | **rMATS** | | | | **Whippet** | | | | **Average** |
| --- | --- | --- | --- | --- | --- | --- | --- | --- | --- | --- | --- | --- |
| *Gene* | *Type* | *Flanks* | *Coordinates* | *FDR* | *PSI Scr* | *PSI AON* | *dPSI* | *Prob* | *PSI Scr* | *PSI AON* | *dPSI* | *dPSI* |
| APP | SE | 21:25880550-25881771, 21:25897573-25897673 | 21:25891722-25891868 | 0 | 1.0 | 0.575 | 0.425 | 1 | 0.99993 | 0.61131 | 0.38862 | 0.40681 |
| **Scrambled AON vs. ATXN3-AON** | | | | **rMATS** | | | | **Whippet** | | | | **Average** |
| *Gene* | *Type* | *Flanks* | *Coordinates* | *FDR* | *PSI Scr* | *PSI AON* | *dPSI* | *Prob* | *PSI Scr* | *PSI AON* | *dPSI* | *dPSI* |
| ATXN3 | SE | 14:92058552-92064414, 14:92080965-92081061 | 14:92070935-92071053 | 0 | 1.0 | 0.605 | 0.395 | 0.979 | 0.98189 | 0.66158 | 0.32031 | 0.357655 |
| **Scrambled AON vs. HTT-AON** | | | | **rMATS** | | | | **Whippet** | | | | **Average** |
| *Gene* | *Type* | *Flanks* | *Coordinates* | *FDR* | *PSI Scr* | *PSI AON* | *dPSI* | *Prob* | *PSI Scr* | *PSI AON* | *dPSI* | *dPSI* |
| HTT | A5SS | 4:3129924-3130047 | 4:3127470-3127604 | 0 | 0.996 | 0.551 | 0.445 | 1 | 0.9886 | 0.66794 | 0.32066 | 0.38283 |

**Table S2 Potentially hybridization-dependent events induced by ATXN3-AON that showed a larger absolute dPSI than the intended event in ATXN3.** SE: exon skipping; A5SS: alternative donor splice site; FDR: false discovery rate; PSI: percent spliced in; dPSI: delta PSI; Scr: scrambled AON; Prob: probability.

| **Scrambled AON vs. ATXN3-AON** | | | | **rMATS** | | | | **Whippet** | | | | **Average** |
| --- | --- | --- | --- | --- | --- | --- | --- | --- | --- | --- | --- | --- |
| *Gene* | *Type* | *Flanks* | *Coordinates* | *FDR* | *PSI Scr* | *PSI AON* | *dPSI* | *Prob* | *PSI Scr* | *PSI AON* | *dPSI* | *dPSI* |
| GOLGA3 | SE | 12:132774496-132775305,12:132777665-132777805 | 12:132776634-132776756 | 0.00 | 1.00 | 0.27 | 0.73 | 1.00 | 1.00 | 0.27 | 0.73 | 0.73 |
| GOLGA3 | SE | 12:132774496-132775305,12:132777665-132777805 | 12:132776958-132777090 | 0.00 | 1.00 | 0.36 | 0.64 | 1.00 | 0.99 | 0.43 | 0.57 | 0.60 |
| HMCN2 | SE | 9:130405954-130406168,9:130408742-130408933 | 9:130407571-130407705 | 0.00 | 0.98 | 0.41 | 0.58 | 1.00 | 0.99 | 0.38 | 0.61 | 0.60 |
| GSDME | SE | 7:24702759-24702833,7:24708126-24708254 | 7:24706184-24706376 | 0.00 | 1.00 | 0.46 | 0.54 | 1.00 | 1.00 | 0.40 | 0.60 | 0.57 |
| GOLGA3 | SE | 12:132774496-132775305,12:132776957-132777090 | 12:132776634-132776756 | 0.00 | 1.00 | 0.69 | 0.31 | 1.00 | 1.00 | 0.27 | 0.73 | 0.52 |
| DAGLB | SE | 7:6425987-6426114,7:6432836-6432959 | 7:6430480-6430606 | 0.00 | 0.99 | 0.47 | 0.53 | 1.00 | 0.97 | 0.48 | 0.49 | 0.51 |
| HNRNPC | SE | 14:21233952-21234229,14:21269297-21269403 | 14:21263311-21263336 | 0.00 | 0.75 | 0.12 | 0.63 | 1.00 | 0.90 | 0.54 | 0.36 | 0.49 |
| RFX4 | SE | 12:106654227-106654351,12:106686883-106687097 | 12:106681993-106682053 | 0.00 | 0.96 | 0.31 | 0.65 | 0.99 | 0.99 | 0.68 | 0.32 | 0.48 |
| RYR3 | SE | 15:33756305-33756373,15:33768657-33768707 | 15:33757475-33757596 | 0.00 | 0.95 | 0.51 | 0.45 | 0.99 | 0.92 | 0.47 | 0.45 | 0.45 |
| FLOT1 | SE | 6:30727708-30728145,6:30730427-30730565 | 6:30730022-30730186 | 0.00 | 1.00 | 0.55 | 0.45 | 1.00 | 1.00 | 0.58 | 0.42 | 0.44 |
| USP48 | SE | 1:21701497-21701602,1:21704261-21704392 | 1:21703512-21703618 | 0.00 | 0.99 | 0.59 | 0.41 | 1.00 | 0.99 | 0.55 | 0.44 | 0.43 |
| DAGLB | SE | 7:6425987-6426114,7:6432836-6432959 | 7:6430480-6430607 | 0.00 | 1.00 | 0.66 | 0.34 | 1.00 | 0.97 | 0.48 | 0.49 | 0.41 |
| DAGLB | SE | 7:6424751-6424835,7:6432836-6432959 | 7:6430480-6430607 | 0.00 | 1.00 | 0.73 | 0.27 | 1.00 | 0.97 | 0.48 | 0.49 | 0.38 |
| PUM1 | SE | 1:30974650-30974802,1:31028795-31028864 | 1:31006994-31007099 | 5.19*10^-15^ | 1.00 | 0.50 | 0.50 | 1.00 | 0.97 | 0.72 | 0.25 | 0.37 |
| GOLGA2 | SE | 9:128259006-128259082,9:128260075-128260189 | 9:128259167-128259390 | 0.00 | 1.00 | 0.51 | 0.49 | 1.00 | 1.00 | 0.74 | 0.25 | 0.37 |
| TRPV1 | A5SS | 17:3585767-3585926 | 17:3588187-3588217 | 0.00 | 1.00 | 0.71 | 0.29 | 1.00 | 0.96 | 0.51 | 0.45 | 0.37 |
| CHMP5 | SE | 9:33267852-33267899,9:33271151-33271223 | 9:33270623-33270716 | 0.00 | 1.00 | 0.62 | 0.38 | 1.00 | 1.00 | 0.66 | 0.34 | 0.36 |
| RFX4 | SE | 12:106654227-106654351,12:106686883-106687097 | 12:106681993-106682054 | 0.00 | 0.99 | 0.59 | 0.40 | 0.99 | 0.99 | 0.68 | 0.32 | 0.36 |
| MECP2 | SE | X:154032206-154032557,X:154097603-154097737 | X:154039792-154039903 | 0.00 | 0.02 | 0.27 | -0.25 | 0.99 | 0.09 | 0.57 | -0.48 | -0.36 |

**Table S3 Shared non-hybridization-dependent events larger than the intended events of the AONs.** SE: exon skipping; dPSI: delta PSI.

|  |  |  |  | **APP-AON** | **ATXN3-AON** | **HTT-AON** |
| --- | --- | --- | --- | --- | --- | --- |
| *Gene* | *Type* | *Flanks* | *Coordinates* | *dPSI* | *dPSI* | *dPSI* |
| ARHGAP29 | SE | 1:94189925-94190083,1:94202543-94202732 | 1:94201720-94201857 | -0.442 | -0.619 | -0.442 |
| CDC16 | SE | 13:114236798-114236896,13:114239349-114239490 | 13:114238990-114239028 | -0.489 | -0.483 | -0.489 |
| CNN3 | SE | 1:94901668-94901785,1:94903121-94903188 | 1:94902121-94902258 | -0.533 | -0.532 | -0.533 |
| IFTAP | SE | 11:36610080-36610239,11:36648015-36650464 | 11:36633284-36633438 | -0.442 | -0.388 | -0.442 |
| INTS6L | SE | X:135575083-135575226,X:135579787-135580162 | X:135577193-135577295 | -0.525 | -0.520 | -0.525 |
| LGR4 | SE | 11:27380639-27380711,11:27382187-27382256 | 11:27380895-27380966 | -0.425 | -0.425 | -0.425 |
| PIKFYVE | SE | 2:208350770-208350947,2:208352653-208352782 | 2:208351352-208351455 | -0.453 | -0.466 | -0.453 |
| PRXL2C | SE | 9:96651389-96651495,9:96654704-96654773 | 9:96651659-96651712 | -0.434 | -0.432 | -0.434 |
| RAPGEF2 | SE | 4:159186641-159186712,4:159210499-159210583 | 4:159193200-159193256 | -0.586 | -0.584 | -0.586 |
| VTI1B | SE | 14:67659730-67659922,14:67674374-67674632 | 14:67662477-67662535 | -0.582 | -0.513 | -0.582 |
| YME1L1 | SE | 10:27136275-27136385,10:27145427-27145590 | 10:27142387-27142485 | -0.556 | -0.457 | -0.556 |
| YY1AP1 | SE | 1:155679408-155679512,1:155688070-155688201 | 1:155680416-155680456 | -0.541 | -0.508 | -0.541 |

**Table S4 Shared splicing events by ATXN3-AON and ATXN3-AON2.** SE: exon skipping; dPSI: delta PSI.

| *Gene* | *Type* | *Flanks* | *Coordinates* | **dPSI C1.1** | **dPSI C1.2** | **dPSI C2** |
| --- | --- | --- | --- | --- | --- | --- |
| BCLAF1 | SE | 6:136261264-136261477,6:136268161-136268339 | 6:136267029-136267175 | 0.205 | -0.210 | -0.252 |
| BECN1 | SE | 17:42814523-42814673,17:42818220-42818415 | 17:42815908-42816054 | 0.237 | 0.210 | 0.324 |
| NUCB1 | SE | 19:48921153-48921324,19:48922317-48923255 | 19:48921869-48921932 | 0.522 | 0.296 | 0.528 |
| SMARCC2 | SE | 12:56164302-56164731,12:56168059-56168194 | 12:56165318-56165699 | 0.468 | 0.281 | 0.519 |
| SGSM2 | SE | 17:2372952-2373081,17:2375491-2375875 | 17:2373331-2373513 | 0.376 | 0.220 | 0.365 |
| VEZF1 | SE | 17:57971551-57974900,17:57980602-57980786 | 17:57979152-57979313 | 0.582 | 0.162 | 0.390 |
