# Supplementary material for: Determining off-target effects of splice-switching antisense oligonucleotides using short read RNAseq in neuronally differentiated human induced pluripotent stem cells
Source: Hum Mol Genet. 2025 Oct 9;34(22):1912–25. doi: 10.1093/hmg/ddaf153 (PMC12581821; doi:10.1093/hmg/ddaf153)
Supplement: Supplementary_file_4_Optimization_ddaf153 [file supplementary_file_4_optimization_ddaf153.pdf]

Supplementary File 4: Optimization of differential splicing detection

We selected rMATS and Whippet as methods to detect differential splicing as rMATS performs well on detection of novel events and the combination of both methods has been shown to improve the FDR and a pipeline to compare the methods is already established. During the setting optimization, the designed event that was validated with PCR was used as positive control and the aim was to focus on robust effects and reduce the noise as much as possible.

rMATS optimization

We started optimization based on the comparison between samples treated with scrambled AON and APP-AON, enabling --*variable-read-length* and --*allow-clipping*. Since we were interested in robust and biologically relevant splicing alterations, we followed the recommendation from Liang et al to change the cutoff splicing difference (*cstat*) value to 5% instead of using the default (0.01%). The change of this setting drastically decreased the number of significant effects (based on junction counts only) from 14957 events to 1540 events. As the AONs could induce the use of an unannotated splice site, we allowed --*novelSS*. (Indeed, the positive effect for the HTT-AON was only detected when novel splice sites were enabled.) When allowing novel splice sites, 3832 significant events were detected for APP-AON. These settings were applied to all three comparisons (Table 1, 'setting 1'). With these settings, about 87% of all reads were used in the analysis. Next, we tested the effect of changing the library type to fr-firststrand, as the data was strand-specific, and setting the anchor length at 15 instead of 1 to reduce false positives. Also, we tried the use of a gtf-file without lowly expressed genes as recommended (1). The positive events were found with all setting variations. We combined the libType *fr*-firststrand, anchor length 15 and use of a filtered gtf-file into 'setting 2' (Table 1).

Table 1 Total and significant events found with use of different settings for the comparison of samples treated with scrambled AON and APP-AON (APP), ATXN3-AON (ATXN3) or HTT-AON (HTT).

APP	Setting 1		Fr-firststrand		Anchor length 15		Filtered GTF		Setting 2	
Type	Total	Sign	Total	Sign	Total	Sign	Total	Sign	Total	Sign
SE	306087	**2476**	304885	**2468**	291634	**2225**	303695	**2460**	288297	**2206**
A5SS	94425	**281**	93943	**272**	86879	**195**	93325	**281**	85419	**190**
A3SS	140043	**344**	139452	**336**	127348	**230**	138821	**340**	125682	**227**
MXE	113743	**624**	112914	**614**	110034	**565**	111226	**589**	106832	**550**
RI	41946	**107**	41749	**107**	39474	**122**	41554	**106**	38876	**119**
ATXN3	Setting 1		Fr-firststrand		Anchor length 15		Filtered GTF		Setting 2	
Type	Total	Sign	Total	Sign	Total	Sign	Total	Sign	Total	Sign
SE	395229	**24742**	393665	**24640**	377224	**22352**	392383	**24614**	373119	**22096**
A5SS	110667	**3409**	110123	**3318**	101570	**2685**	109516	**3350**	99980	**2543**
A3SS	165365	**3769**	164627	**3688**	149712	**2637**	164035	**3745**	147797	**2507**
MXE	175568	**9467**	174312	**9401**	170113	**8352**	171819	**9194**	165286	**8057**
RI	48434	**3016**	48185	**2749**	45689	**2706**	48008	**3005**	44994	**2417**
HTT	Setting 1		Fr-firststrand		Anchor length 15		Filtered GTF		Setting 2	
Type	Total	Sign	Total	Sign	Total	Sign	Total	Sign	Total	Sign

SE	338986	**4232**	337540	**4196**	322618	**3707**	336172	**4196**	318619	**3644**
A5SS	99635	**501**	99192	**495**	91706	**375**	98507	**490**	90223	**351**
A3SS	150554	**587**	149870	**578**	136884	**399**	149298	**585**	135066	**385**
MXE	132595	**1100**	131739	**1090**	128597	**943**	129479	**1070**	124778	**913**
RI	43629	**247**	43436	**208**	41058	**208**	43224	**243**	40449	**183**

Next, we compared the detection of the positive events between setting 1 and 2 after filtering the rMATS results on informative reads present for each event (at least 10 reads for each sample, Figure 1). Whereas the detection of the positive APP- and HTT-AON effect seemed to be similar, the detection of the ATXN3-AON event was more specific.

APP - setting 1

```
> df[match(df.filtered_APP_ID,df$ID),]
           ID          GeneID geneSymbol   chr strand exonStart_0base  exonEnd upstreamES upstreamEE downstreamES downstreamEE   ID.1                            IJC_SAMPLE_1  SJC_SAMPLE_1
14604   16924 ENSG00000142192        APP chr21      -        25891721 25891868   25880534   25881771     25897572     25897673  16924 15598,20116,24216,15447,15679,18096,16786 0,0,0,3,0,0,0
158342 182700 ENSG00000142192        APP chr21      -        25891721 25891855   25880534   25881771     25897572     25897983 182700    7196,9121,10885,7209,7154,8576,7648 0,0,0,3,0,0,0
158343 182701 ENSG00000142192        APP chr21      -        25891721 25891865   25880534   25881771     25897572     25897983 182701    7563,9675,11532,7610,7612,9076,8172 0,0,0,3,0,0,0
158346 182704 ENSG00000142192        APP chr21      -        25891721 25891902   25880534   25881771     25897572     25897983 182704    7615,9783,11657,7678,7655,9183,8261 0,0,0,3,0,0,0
158348 182707 ENSG00000142192        APP chr21      -        25891814 25891868   25881609   25881771     25897572     25897673 182707     2267,2702,3645,2015,2084,2233,2531 0,0,0,3,0,0,0
                          IJC_SAMPLE_2                     SJC_SAMPLE_2 IncFormLen SkipFormLen PValue FDR                      IncLevel1                             IncLevel2 IncLevelDifference
14604  7685,8470,8588,7765,11659,10557 3002,3027,3749,3368,3982,3744        296         149      0   0   1.0,1.0,1.0,1.0,1.0,1.0,1.0 0.563,0.585,0.536,0.537,0.596,0.587              0.433
158342   3568,3977,3990,3578,5476,4826 3002,3027,3749,3368,3982,3744        283         149      0   0 1.0,1.0,1.0,0.999,1.0,1.0,1.0  0.385,0.409,0.359,0.359,0.42,0.404              0.611
158343   3787,4205,4214,3771,5796,5107 3002,3027,3749,3368,3982,3744        293         149      0   0 1.0,1.0,1.0,0.999,1.0,1.0,1.0  0.391,0.414,0.364,0.363,0.425,0.41              0.605
158346   3854,4235,4249,3798,5840,5160 3002,3027,3749,3368,3982,3744        298         149      0   0 1.0,1.0,1.0,0.999,1.0,1.0,1.0 0.391,0.412,0.362,0.361,0.423,0.408              0.607
158348   1012,1099,1085,1055,1481,1534 3002,3027,3749,3368,3982,3744        203         149      0   0 1.0,1.0,1.0,0.998,1.0,1.0,1.0  0.198,0.21,0.175,0.187,0.214,0.231              0.797
```

APP- setting 2

```
> df[match(df.filtered_APP_ID,df$ID),]
           ID          GeneID geneSymbol   chr strand exonStart_0base  exonEnd upstreamES upstreamEE downstreamES downstreamEE   ID.1                            IJC_SAMPLE_1  SJC_SAMPLE_1
31195   36243 ENSG00000142192        APP chr21      -        25891721 25891868   25880534   25881771     25897572     25897673  36243 13005,16448,19785,12638,12813,14977,13542 0,0,0,3,0,0,0
170235 196922 ENSG00000142192        APP chr21      -        25891721 25891855   25880534   25881771     25897572     25897983 196922    6572,8294,9900,6497,6496,7758,6871 0,0,0,3,0,0,0
170236 196923 ENSG00000142192        APP chr21      -        25891721 25891865   25880534   25881771     25897572     25897983 196923    6624,8365,9994,6550,6545,7857,6925 0,0,0,3,0,0,0
170238 196926 ENSG00000142192        APP chr21      -        25891721 25891902   25880534   25881771     25897572     25897983 196926    6624,8366,9994,6550,6545,7856,6925 0,0,0,3,0,0,0
170240 196929 ENSG00000142192        APP chr21      -        25891814 25891868   25881609   25881771     25897572     25897673 196929    1221,1396,1906,1086,1057,1251,1392 0,0,0,3,0,0,0
                        IJC_SAMPLE_2                  SJC_SAMPLE_2 IncFormLen SkipFormLen PValue FDR                      IncLevel1                             IncLevel2 IncLevelDifference
31195  6365,6959,7055,6357,9716,8632 2410,2431,2950,2646,3128,2968        242         121      0   0   1.0,1.0,1.0,1.0,1.0,1.0,1.0 0.569,0.589,0.545,0.546,0.608,0.593              0.425
170235 3281,3613,3618,3213,4953,4378 2410,2431,2950,2646,3128,2968        242         121      0   0 1.0,1.0,1.0,0.999,1.0,1.0,1.0  0.405,0.426,0.38,0.378,0.442,0.424              0.591
170236 3309,3633,3628,3236,5003,4423 2410,2431,2950,2646,3128,2968        242         121      0   0 1.0,1.0,1.0,0.999,1.0,1.0,1.0 0.407,0.428,0.381,0.379,0.444,0.427              0.589
170238 3309,3633,3628,3236,5003,4423 2410,2431,2950,2646,3128,2968        242         121      0   0 1.0,1.0,1.0,0.999,1.0,1.0,1.0 0.407,0.428,0.381,0.379,0.444,0.427              0.589
170240       557,572,561,582,842,827 2410,2431,2950,2646,3128,2968        175         121      0   0 1.0,1.0,1.0,0.996,1.0,1.0,1.0  0.138,0.14,0.116,0.132,0.157,0.162              0.859
```

ATXN3 - setting 1

```
> df[match(df.filtered_ATXN3_ID,df$ID),]
           ID          GeneID geneSymbol   chr strand exonStart_0base  exonEnd upstreamES upstreamEE downstreamES downstreamEE   ID.1           IJC_SAMPLE_1  SJC_SAMPLE_1
70587   80832 ENSG00000066427      ATXN3 chr14      -        92070934 92071053   92049610   92049814     92080964     92081061  80832  43,44,65,47,47,59,40 0,0,0,0,0,0,0
70588   80833 ENSG00000066427      ATXN3 chr14      -        92070934 92071053   92050340   92050536     92080964     92081061  80833  43,44,62,48,48,59,40 0,0,0,0,0,0,0
70589   80834 ENSG00000066427      ATXN3 chr14      -        92070934 92071053   92058551   92064414     92080964     92081061  80834 88,76,121,87,65,97,67 0,0,0,0,0,0,0
266276 298014 ENSG00000066427      ATXN3 chr14      -        92070934 92071049   92058551   92064414     92080964     92081061 298014  43,24,57,41,12,32,24 0,0,0,0,0,0,0
             IJC_SAMPLE_2      SJC_SAMPLE_2 IncFormLen SkipFormLen       PValue          FDR                   IncLevel1                            IncLevel2 IncLevelDifference
70587     36,20,12,17,12,9  24,0,1,0,20,14        268         149 3.780468e-04 6.978752e-03 1.0,1.0,1.0,1.0,1.0,1.0,1.0       0.455,1.0,0.87,1.0,0.25,0.263              0.360
70588      36,20,12,17,9,9   4,12,2,3,1,12        268         149 8.707231e-08 2.356444e-06 1.0,1.0,1.0,1.0,1.0,1.0,1.0 0.833,0.481,0.769,0.759,0.833,0.294              0.339
70589   48,45,28,35,27,17 10,18,9,23,4,12        268         149 0.000000e+00 0.000000e+00 1.0,1.0,1.0,1.0,1.0,1.0,1.0  0.727,0.582,0.634,0.458,0.79,0.441              0.395
266276    0,17,11,14,15,6 10,18,9,23,4,12        264         149 0.000000e+00 0.000000e+00 1.0,1.0,1.0,1.0,1.0,1.0,1.0    0.0,0.348,0.408,0.256,0.679,0.22              0.681
```

ATXN3 - setting 2

```
> df[match(df.filtered_ATXN3_ID,df$ID),]
         ID          GeneID geneSymbol   chr strand exonStart_0base  exonEnd upstreamES upstreamEE downstreamES downstreamEE  ID.1          IJC_SAMPLE_1  SJC_SAMPLE_1
72478 83829 ENSG00000066427      ATXN3 chr14      -        92070934 92071053   92058551   92064414     92080964     92081061 83829 82,62,109,70,40,74,59 0,0,0,0,0,0,0
           IJC_SAMPLE_2 SJC_SAMPLE_2 IncFormLen SkipFormLen PValue FDR                   IncLevel1                            IncLevel2 IncLevelDifference
72478 28,35,20,29,24,10 6,15,8,9,4,6        240         121      0   0 1.0,1.0,1.0,1.0,1.0,1.0,1.0 0.702,0.541,0.558,0.619,0.752,0.457              0.395
```

HTT - setting 1

```
> df[match(df.filtered_HTT_ID,df$ID),]
         ID          GeneID geneSymbol  chr strand longExonStart_0base longExonEnd shortES shortEE flankingES flankingEE  ID.1              IJC_SAMPLE_1  SJC_SAMPLE_1
46298 50742 ENSG00000197386        HTT chr4      +             3127263     3127604 3127263 3127469    3129923    3130047 50742 109,131,131,71,61,100,102 0,0,1,0,0,1,0
                 IJC_SAMPLE_2              SJC_SAMPLE_2 IncFormLen SkipFormLen PValue FDR                       IncLevel1                                IncLevel2 IncLevelDifference
46298 96,107,81,90,56,75,54 37,48,55,26,33,59,22        284         149      0   0 1.0,1.0,0.986,1.0,1.0,0.981,1.0 0.576,0.539,0.436,0.645,0.471,0.4,0.563              0.477
```

HTT - setting 2

```
> df[match(df.filtered_HTT_ID,df$ID),]
         ID          GeneID geneSymbol  chr strand longExonStart_0base longExonEnd shortES shortEE flankingES flankingEE  ID.1          IJC_SAMPLE_1  SJC_SAMPLE_1
28102 33076 ENSG00000197386        HTT chr4      +             3127263     3127604 3127263 3127469    3129923    3130047 33076 86,105,93,58,51,73,85 0,0,0,0,0,1,0
               IJC_SAMPLE_2           SJC_SAMPLE_2 IncFormLen SkipFormLen PValue FDR                     IncLevel1                               IncLevel2 IncLevelDifference
28102 68,84,56,71,47,59,48 19,42,40,15,21,33,16        242         121      0   0 1.0,1.0,1.0,1.0,1.0,0.973,1.0 0.642,0.5,0.412,0.703,0.528,0.472,0.6              0.445
```

Figure 1 Detection of positive events using setting 1 or 2.

Whippet optimization

The pipeline of Whippet consists of three scripts: making an index, quantifying each sample and calculating the delta PSI between two sample groups. The optimization of Whippet was aimed at getting results comparable to the results of rMATS while adjusting the settings to the tool's own needs to get robust results.

To allow detection of novel events, we supplied a merged bam-file containing the samples treated with scrambled AON and a targeting AON (APP-, ATXN3- or HTT-AON) to include de novo splice sites in the index. The setting *–suppress-low-tsl* was enabled to reduce noise. Two settings could be adjusted influencing the detection of novel splice sites: the minimum number of reads supporting a splice site from BAM to be included in the index (*--bam-min-reads*) and whether splicing reads with two de novo splice sites were allowed (*--bam-both-novel*). As expected, the number of de novo

splice sites was higher when --*bam-both-novel* was enabled and decreased with the use of a higher minimum reads (Table 2). For all options tried, the novel splice site introduced by HTT-AON could be detected.

Table 2 Detection of splice-sites in the GTF file and based on the BAM-file using different settings.

**Bam-min-reads**	**Bam-both-novel**	**+**	**-**
**2**	Scrapp	187150 BAM<br>533929 GTF	78624 BAM<br>533929 GTF
**2**	Scrhtt	190956 BAM<br>533929 GTF	80555 BAM<br>533929 GTF
**3**	Scrapp	124833 BAM<br>533929 GTF	52742 BAM<br>533929 GTF
**6**	Scrapp	72367 BAM<br>533929 GTF	26848 BAM<br>533929 GTF
**6**	Scrhtt	73502 BAM<br>533929 GTF	27571 BAM<br>533929 GTF
**6**	Scratxn3	85369 BAM<br>533929 GTF	34813 BAM<br>533929 GTF

For the final index, we enabled --*bam-both-novel* to include all potential AON-related effects and we chose a minimum reads of 6, since each group had at least 6 samples. Also, we used the same filtered GTF-file as used for setting 2 of rMATS.

However, with these settings, the positive event for ATXN3-AON could not be found. The only event found for exon 10 contained only a part of the exon and was classified as tandem alternative polyadenylation site (use of exon 10 as last exon instead of exon 11), which is not the case, with a low probability and very small deltaPSI. Also with adjusting the minimum reads threshold in the script to calculate the deltaPSI from default (5) to 1, since the expression of ATXN3 was low, did not change the outcome. The first nucleotides of exon 10 (92070935:92071037) were not quantified by default (node without a simple type, NA) which could contribute to the failure to detect the exon skip. As the only remedy to change this is simplifying the input reference annotation, we looked at the number of different isoforms annotated for ATXN3. Compared to APP (20 isoforms) and HTT (23 isoforms), the number of ATXN3 isoforms (54) is indeed quite high. The high number of isoforms might especially be complicated as Whippet distinguishes a larger number of event types compared to rMATS. In combination with the low expression of ATXN3, this might complicate proper splice detection. Therefore, we followed the advice to use a simplified reference annotation and tried the use of isoforms annotated with gencode-basic only. Now, the skip of exon 10 was finally detected. To be consistent, we used the gencode-basic gtf for all three comparisons.

Assessing overlap

To see whether the use of a gtf subset (gencode-basic) improved the performance of Whippet in particular or whether a consistent use of the same gtf as input for both Whippet and rMATS was preferable, we compared the overlap of the significant results for both tools with different input gtf-files (Table 3). In the overlap, only the event types shared by both tools are present: SE, A5SS, A3SS and RI. The overlap of significant events is only about 25%, which is consistent with literature (2). The use of a basic gtf-file in Whippet increases the overlap with rMATS. For rMATS, the use of a basic gtf-file does not change much in the overlap. Especially considering the larger events with an

absolute deltaPSI >0.3 in both tools, the overlap is the same. This is not surprising as splice sites that have been removed from the gtf-file can still be detected as novel splice site as de novo site detection is enabled. To focus on robust findings, we chose to use the basic gtf-file for rMATS as well.

Table 3 Overlap of significant events found by rMATS or Whippet while using the gtf-file filtered on expression levels or also filtered for genes annotated as gencode-basic ('basic gtf'). The significant events obtained for the different comparisons are listed in the first column for rMATS and the second row for Whippet (containing only the four event types occuring in both tools). The overlap presented is the 1) number of overlapping events, 2) percentage of the significant events found by the tools on their own and 3) the number of overlapping events with a deltaPSI >0.3 in both tools.

	Whippet			***Whippet – basic***		
rMATS	**APP: 3029**	**ATXN3: 20003**	**HTT: 4334**	**APP: 2703**	**ATXN3: 18703**	**HTT: 4107**
**APP: 1813**	436<br>14-24%<br>67			467<br>17-26%<br>72		
**ATXN3: 22671**		5911<br>26-30%<br>747			6088<br>27-33%<br>789	
**HTT: 3225**			768<br>18-24%<br>55			839<br>20-26%<br>59
rMATS – basic gtf				***Whippet – basic***		
**APP: 1738**				463<br>17-27%<br>73		
**ATXN3: 21300**					6012<br>28-32%<br>788	
**HTT: 3118**						843<br>21-27%<br>60

Finally, we wondered whether disabling the setting *–bam-both-novel* would improve the performance of Whippet as the tool's authors have shown that this function increases the FDR and our main focus is on hybridization-dependent effects and the binding of an AON is more likely to induce one novel splice site. Hence, we run the script for Whippet again now without *–bam-both-novel*, both using the 'normal' gtf as the basic gtf, and compared the overlap with rMATS (Table 4). Although there were less significant events identified by Whippet when disabling *–bam-both-novel*, the overlap with the rMATS results was higher. Thus, we chose to disable this option while generating the index for Whippet. The numbers of novel splice sites detected in the BAM files with or without *–bam-both-novel* for the normal gtf or basic gtf are listed in Table 5.

*Table 4 Overlap of significant events found by rMATS or Whippet (disabled –*bam-both-novel*) while using the gtf-file filtered on expression levels or also filtered for genes annotated as gencode-basic ('basic gtf'). The significant events obtained for the different comparisons are listed in the first column for rMATS and the second row for Whippet (containing only the four event types occuring in both tools). The overlap presented is the 1) number of overlapping events, 2) percentage of the*

significant events found by the tools on their own and 3) the number of overlapping events with a deltaPSI >0.3 in both tools.

	Whippet – bbn off			***Whippet – basic – bbn off***		
rMATS	**APP: 2720**	**ATXN3: 18015**	**HTT: 3959**	**APP: 2445**	**ATXN3: 16745**	**HTT: 3712**
**APP: 1813**	462<br>17-25%<br>69			491<br>20-27%<br>74		
**ATXN3: 22671**		6136<br>27-34%<br>764			6280<br>28-38%<br>819	
**HTT: 3225**			816<br>21-25%<br>63			860<br>23-27%<br>63
rMATS – basic gtf				***Whippet – basic***		
**APP: 1738**				489<br>20-28%<br>75		
**ATXN3: 21300**					6230<br>29-37%<br>820	
**HTT: 3118**						867<br>23-28%<br>64

Table 5 Detection of splice-sites in the GTF file and based on the BAM-file using the normal or basic GTF file, with or without –bam-both-novel. All indexes are based on –bam-min-reads of 6.

**GTF (filtered)**	**Bam-both-novel**	**+**	**-**
**Normal**	Scrapp	61618 BAM<br>429970 GTF	24243 BAM<br>429970 GTF
**Normal**	Scratxn3	73623 BAM<br>429970 GTF	31791 BAM<br>429970 GTF
**Normal**	Scrhtt	62678 BAM<br>429970 GTF	24890 BAM<br>429970 GTF
**Basic**	Scrapp	60291 BAM<br>384618 GTF	28513 BAM<br>384618 GTF
**Basic**	Scratxn3	75158 BAM<br>384618 GTF	36392 BAM<br>384618 GTF
**Basic**	Scrhtt	61384 BAM<br>384618 GTF	29181 BAM<br>384618 GTF

Final settings

The final settings have been described in the method section. Table 6 lists the number of events obtained using the basic gtf-file for both rMATS and Whippet for all event types detected by the tools, whereas the overlap of the event types assessed by both tools is listed in Table 7.

Table 6 Final number of (significant) results obtained by rMATS (after filtering results on read count) and Whippet per event type.

rMATS	APP		ATXN3		HTT	
Type	Total	Sign	Total	Sign	Total	Sign
SE	184479	**1527**	229819	**17347**	200313	**2746**
A5SS	50738	**88**	56311	**1503**	52353	**145**
A3SS	76760	**80**	85336	**1320**	80983	**165**
MXE	69435	**414**	103745	**6902**	82235	**636**
RI	17039	**43**	18880	**1130**	17329	**62**
Whippet	APP		ATXN3		HTT	
Type	Total	Sign	Total	Sign	Total	Sign
AA	8579	**210**	9205	**1152**	8535	**223**
AD	16126	**350**	19842	**3462**	16682	**375**
AF	3005	**32**	2943	**222**	3003	**71**
AL	1035	**23**	951	**75**	1005	**33**
CE	146922	**1822**	148228	**11629**	146730	**3018**
RI	2715	**63**	2838	**502**	2717	**96**
TE	27079	**515**	27079	**1256**	26965	**513**
TS	20169	**277**	19852	**454**	20064	**351**

Table 7 Number of significant results obtained by rMATS and Whippet and overlap of significant results per event type. SE/CE: exon skipping; A5SS/AD: alternative splice donor site; A3SS/AA: alternative splice acceptor site; RI: retained intron.

	**APP-AON**			**ATXN3-AON**			**HTT-AON**		
Type	*rMATS*	*Whippet*	***Overlap***	*rMATS*	*Whippet*	***Overlap***	*rMATS*	*Whippet*	***Overlap***
SE/CE	1527	1822	468	17347	11629	5741	2746	3018	842
A5SS/AD	88	350	9	1503	3462	286	145	375	14
A3SS/AA	80	210	10	1320	1152	142	165	223	9
RI	43	63	2	1130	502	61	62	96	2

References

1. Jiang M, Zhang S, Yin H, Zhuo Z, Meng G. A comprehensive benchmarking of differential splicing tools for RNA-seq analysis at the event level. Briefings in bioinformatics. 2023;24(3).

2. Olofsson D, Preußner M, Kowar A, Heyd F, Neumann A. One pipeline to predict them all? On the prediction of alternative splicing from RNA-Seq data. Biochemical and biophysical research communications. 2023;653:31-7.
